# An interactive genome browser of association results from the UK10K cohorts project

**DOI:** 10.1093/bioinformatics/btv491

**Published:** 2015-08-26

**Authors:** Matthias Geihs, Ying Yan, Klaudia Walter, Jie Huang, Yasin Memari, Josine L. Min, Daniel Mead, UK10K Consortium, Tim J. Hubbard, Nicholas J. Timpson, Thomas A. Down, Nicole Soranzo

**Affiliations:** ^1^Wellcome Trust Sanger Institute, Genome Campus, Hinxton CB10 1HH, UK,; ^2^MRC Integrative Epidemiology Unit, University of Bristol, Oakfield Grove, Bristol, UK,; ^3^Department of Medical and Molecular Genetics, Division of Genetics and Molecular Medicine, King's College London School of Medicine, Guy's Hospital, London SE1 9RT, UK,; ^4^EMBL-EBI, Hinxton CB10 1SD, UK and; ^5^Department of Haematology, University of Cambridge, Cambridge CB2 1TN, UK

## Abstract

**Summary:** High-throughput sequencing technologies survey genetic variation at genome scale and are increasingly used to study the contribution of rare and low-frequency genetic variants to human traits. As part of the Cohorts arm of the UK10K project, genetic variants called from low-read depth (average 7×) whole genome sequencing of 3621 cohort individuals were analysed for statistical associations with 64 different phenotypic traits of biomedical importance. Here, we describe a novel genome browser based on the Biodalliance platform developed to provide interactive access to the association results of the project.

**Availability and implementation**: The browser is available at http://www.uk10k.org/dalliance.html. Source code for the Biodalliance platform is available under a BSD license from http://github.com/dasmoth/dalliance, and for the LD-display plugin and backend from http://github.com/dasmoth/ldserv.

**Contact**: ns6@sanger.ac.uk or thomas@biodalliance.org

**Supplementary information**: Supplementary data are available at *Bioinformatics* online.

## 1 Introduction

Rare and low-frequency genetic variants play an important role in determining population variance of complex traits and disease; however, until recently their systematic evaluation has been beyond the reach of empirical population-based genetic studies.

**Fig. 1.**
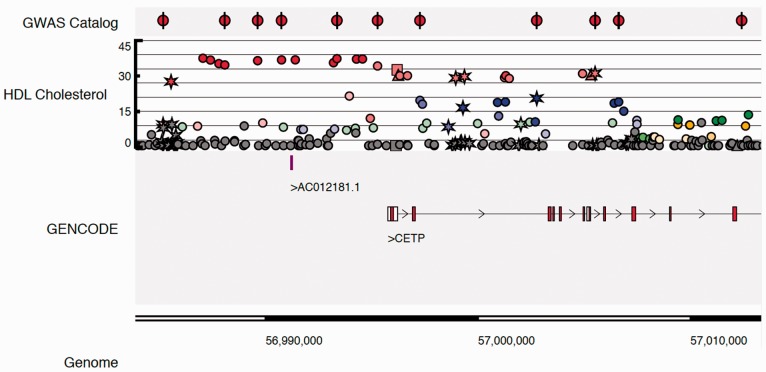
Example screenshot of the UK10K Genome Browser. The panel shows a 30-kb region of the human genome where UK10K SNPs associate with HDL Cholesterol with high *P*-values around the CETP gene. Colouring identifies groups of independent SNPs

The UK10K project was designed to characterize rare and low-frequency variation in the UK genome wide, and study its contribution to a broad spectrum of biomedically relevant quantitative traits and diseases with different predicted genetic architectures. The data generated by the different arms of the UK10K project and their use are described elsewhere (UK10K Consortium, 2015). Here, we describe the development of a novel browser for genetic association data based on the Biodalliance platform (Down *et al.,* 2011), designed to facilitate the retrieval of genotype–phenotype association results from the UK10K-cohorts arm of the project, and their visualization in the context of different annotation features (Fig. 1). In particular, we developed a novel interactive display for genetic variants showing both the strength of association with a trait, and the pattern of linkage disequilibrium (LD) within the cohort.

## 2 Implementation

Biodalliance is a pure JavaScript genome browser, using HTML5 canvas for displays. Biodalliance’s preferred approach for fetching data is to use indexed binary files such as bigWig or bigBed files (Kent *et al.,* 2010) which can be made available from normal web-servers and are frequently organized as track-hubs (Raney *et al.,* 2014). Because Biodalliance is pure JavaScript it can be embedded in any normal webpage making it easy to create custom browsers such as presented here.

However, creating a display showing genetic association data posed additional challenges. Firstly, there is no perfect file format for representing the association results themselves (which comprise both a *P*-value from the association test and additional information about the SNP). Secondly, our desire to present LD data in a flexible, interactive manner does not fit well with the usual indexed-file approach of retrieving a single block of data corresponding to some range of genomic coordinates.

To represent the association results, we used a pair of binary files. Firstly, a bigWig file contains the association test result (expressed as a log *P*-value) for each position in the genome where a variant was tested. A bigBed file containing the variant ID and other information such as SNP consequence predictions supplements this. We added support in the core Biodalliance code for merging multiple data sources into a single track. The Biodalliance renderer and stylesheet system runs on this merged dataset, so in our association tracks we can show association score (from a bigWig file) as the *y*-axis position of the feature, but choose a point style based on the SNP consequence (from the bigBed file). The *y*-axis encoding had the added benefit of enabling a feature in the browser to search for the next variant in the genome above an association score threshold (exploiting information encoded in the reduced resolution views of bigWig files) or by identifier (using a name index in the bigBed file).

Dynamic display of LD information poses a different challenge since all-against-all data are unwieldy but we do not know in advance which variants users might want to select as reference. One option would be to create a Biodalliance plug-in that calculates LD on the client side. However, this would require complete genotype data for the cohort, which is also very large, and would raise data security concerns. Instead, we developed a simple server component for LD calculation on the fly for a selected (or multiple) reference variant(s), allowing the genotype data itself to remain securely on the server. We also developed Biodalliance plug-ins for selecting reference variants, and for communicating with the LD server. Once again, the capability to merge results from multiple data sources proved useful, and the LD scores simply get merged into the rest of the feature data before it is passed to the Biodalliance renderer.

Having extended the Biodalliance code in this way, bigWig and files containing *P*-values were prepared for each combination of the traits in Supplementary Appendix S1, and the statistical tests in Supplementary Appendix S2. A description of the statistical tests implemented is given in Supplementary Appendix 3. The large number of resulting files were organized in a track-hub, which the UK10K Genome Browser was configured to use by default. The browser functionality is described in Supplementary Appendix 4. A reference summary of test statistics, and key navigation functions, is given in Supplementary Appendix 5. Finally, a step-by-step user tutorial to the browser is also provided (Supplementary Appendix 6).

## 3 Results

The UK10K Genome Browser exploits the key basic features of the Biodalliance Genome Browser. A number of custom features were specifically designed to aid navigation and interpretation of the UK10K-cohorts data. First, the genome browser supports dynamic estimation of local LD statistics (i.e. the metric *r*^2^) for inferring statistical independence between local association signals and one or more SNP(s) of interest. Calculations are performed in real time using the UK10K-cohorts sequence data. Second, it allows exporting of local association results into high-quality images (in scalable vector graphics, svg) that can be used in publications. Finally, it provides dynamic annotation of each SNP, including information on minor allele frequency, SNP quality metrics, as well as links to SNP annotation resources such as dbSNP and functional genome annotation track-hubs such as those generated by the ENCODE (Encode Project Consortium *et al.,* 2012), NIH Roadmap (Roadmap Epigenomics Consortium *et al.*, 2015) and BLUEPRINT (Adams *et al.,* 2012).

## 4 Conclusions

In summary, the UK10K browser provides an intuitive and efficient platform to access association statistics for common, low-frequency and rare variants against a large number of human phenotypic traits. As efforts progress to systematically map the contribution of human genetic variation to healthy and disease phenotypes, and to integrate it with genome functional resources, the development of platforms like ours will become essential enabling instruments for the integration and cross-validation of genetic discoveries within the scientific community.

## Supplementary Material

Supplementary Data
