# Supplementary material for: An interactive genome browser of association results from the UK10K cohorts project
Source: Bioinformatics. 2015 Aug 26;31(24):4029–31. doi: 10.1093/bioinformatics/btv491 (PMC4673976; doi:10.1093/bioinformatics/btv491)
Supplement: Supplementary Data [file supp_31_24_4029__index.html]

An interactive genome browser of association results from the UK10K cohorts project — An interactive genome browser of association results from the UK10K cohorts project — Supplementary Data 

# An interactive genome browser of association results from the UK10K cohorts project

## Supplementary Data

files

- Supplementary Data - docx file
