# Supplementary material for: An interactive genome browser of association results from the UK10K cohorts project
Source: Bioinformatics. 2015 Aug 26;31(24):4029–31. doi: 10.1093/bioinformatics/btv491 (PMC4673976; doi:10.1093/bioinformatics/btv491)

| Summary Information  An interactive genome browser of association results from the UK10K cohorts project  Matthias Geihs ^1^, Ying Yan ^1,5^, Klaudia Walter ^1^, Jie Huang ^1^, Yasin Memari ^1^, Josine Min ^2^, Daniel Mead ^1^, UK10K Project, Tim Hubbard ^1,3^, Nicholas Timpson ^2^, Thomas Down ^2,4,*^, Nicole Soranzo ^1,5,*^  ^1^ Wellcome Trust Sanger Institute, Genome Campus, Hinxton, CB10 1HH, UK  ^2^ MRC Integrative Epidemiology Unit at the University of Bristol, University of Bristol, Oakfield House, Oakfield Grove, Bristol, UK  ^3^ King’s College London  ^4^ EMBL-EBI, Genome Campus, Hinxton, CB10 1SD, UK  ^5^ Department of Haematology, University of Cambridge, Cambridge CB2, UK  Received on XXXXX; revised on XXXXX; accepted on XXXXX  Associate Editor: XXXXXXX |
| --- |

**Appendix 1. Phenotypic traits investigated in the UK10K cohort project**

| **Category** | **Name** | **TwinsUK** | **ALSPAC** | **Total** |
| --- | --- | --- | --- | --- |
| Obesity/anthropometry | Body mass index (BMI) | 1,747 | 1,791 | 3,538 |
|  | Height (HT) | 1,747 | 1,794 | 3,541 |
|  | Weight (WT) | 1,747 | 1,812 | 3,559 |
|  | Hip circumference (HIP)* | 1,266 | 1,808 | 3,074 |
|  | Waist circumference (WST)* | 1,265 | 1,807 | 3,072 |
|  | Waist hip ratio (WHR)* | 1,265 | 1,806 | 3,071 |
|  | Total fat mass (TFM) | 1,716 | 1,683 | 3,399 |
|  | Total lean mass (TLM) | 1,716 | 1,683 | 3,399 |
|  | Trunk fat mass (TRFM) | 1,514 | 1,683 | 3,197 |
|  | Forearm length (FAL) | - | 1,760 | 1,760 |
|  | Head circumference (HCRF) | - | 1,762 | 1,762 |
|  | Leg length (LL) | - | 1,764 | 1,764 |
|  | Sitting height (SHT) | - | 1,764 | 1,764 |
|  | Upperarm length (UAL) | - | 1,762 | 1,762 |
|  | Adiponectin (ADIPO) | 864 | 1,461 | 2,325 |
|  | Leptin (LEPTN) | 958 | 1,459 | 2,417 |
| Diabetes Biochemistry | Glucose (GLU)* | 1,701 | 1,224 | 2,925 |
|  | HOMA-B (HOMA-B)* | 1,669 | 1,219 | 2,888 |
|  | HOMA-IR (HOMA-IR)* | 1,577 | 1,219 | 2,796 |
|  | Insulin (INS)* | 1,676 | 1,220 | 2,896 |
| Heart function | Heart rate (HRT) | 1,385 | 1,590 | 2,975 |
| CVD hypertension | Diastolic blood pressure (DBP) | 1,536 | 1,773 | 3,309 |
|  | Systolic blood pressure (SBP) | 1,536 | 1,773 | 3,309 |
| CVD Biochemistry | High density lipoprotein cholesterol (HDL) | 1,713 | 1,497 | 3,210 |
|  | Low density lipoprotein cholesterol (LDL) | 1,696 | 1,495 | 3,191 |
|  | Total cholesterol (TC) | 1,711 | 1,495 | 3,206 |
|  | Triglycerides (TG) | 1,705 | 1,497 | 3,202 |
|  | Very low density lipoprotein (VLDL) | 1,700 | 1,497 | 3,197 |
|  | Apolipoprotein A1 (ApoA1) | 1,449 | 1,465 | 2,914 |
|  | Apolipoprotein B (ApoB) | 1,443 | 1,468 | 2,911 |
|  | Homocysteine (HCY) | 1,279 | - | 1,372 |
|  | HsCRP (CRP) | 879 | 1,167 | 2,046 |
| Blood Biochemistry | Hemoglobin (HGB) | 1,553 | 1,524 | 3,077 |
|  | Mean corpuscular hemoglobin (MCH) | 1,549 | - | 1,549 |
|  | Mean corpuscular hemoglobin concentration (MCHC) | 942 | - | 942 |
|  | Mean corpuscular volume (MCV) | 1,548 | - | 1,548 |
|  | Packed cell volume (PCV) | 1,555 | - | 1,555 |
|  | Platelet counts (PLT) | 1,553 | - | 1,553 |
|  | Red blood cell counts (RBC) | 1,561 | - | 1,561 |
|  | White blood cell counts (WBC) | 1,551 | - | 1,551 |
|  | Interleukin 6 (IL6) | - | 1,480 | 1,480 |
| Liver Function | Albumin (ALB) | 1,713 | - | 1,713 |
|  | Alkaline phosphatase (ALP) | 1,702 | - | 1,702 |
|  | Bilirubin (BIL) | 1,702 | - | 1,702 |
|  | Gamma glutamyl transpeptidase (GGT) | 1,699 | - | 1,699 |
| Renal Function | Bicarbonate (BIC) | 1,714 | - | 1,714 |
|  | Creatinine (CRT) | 1,707 | - | 1,707 |
|  | Phosphate (PHPT) | 1,392 | - | 1,392 |
|  | Sodium (SOD) | 1,683 | - | 1,683 |
|  | Urea (UR) | 1,697 | - | 1,697 |
|  | Uric acid (UA) | 1,305 | - | 1,305 |
| Lung Function | FEV/FVC ratio (FEV1-FVC) | 1,676 | 1,604 | 3,280 |
|  | Forced Vital Capacity (FVC) | 1,679 | 1,606 | 3,285 |
|  | Forced Expiratory Volume (FEV1) | 1,681 | 1,606 | 3,287 |
| Birth | Birth weight (BWT) | - | 1,691 | 1,691 |
|  | Birth length (BL) | - | 1,137 | 1,137 |
|  | Gestational age (GA) | - | 1,712 | 1,712 |
|  | Ponderal index (PI) | - | 1,122 | 1,122 |
|  | Placental weight (PLWT) | - | 703 | 703 |
| Dynamic | Grip strength (GRP) | 1,514 | 1,682 | 3,196 |
|  | Ever broken bone** | - | 1,756 | 1,756 |
|  | Eye preference** | - | 1,671 | 1,671 |
|  | Handedness tasks** | - | 1,700 | 1,700 |
|  | Handedness drawing** | - | 1,676 | 1,676 |

** Not in browser

**Appendix 2. List of statistical tests implemented and browser nomenclature**

| **Track name** | **Test** | **Variant selection strategy** | **Windows and variants tested** |
| --- | --- | --- | --- |
| **Single-variant association tests** |  |  |  |
| SIngle_variant | Linear regression | Single-variant tests for MAF>0.1% | 14,196,778  (median = 13,933,511,  range = [13,450,148-15,336,389]) |
| **Exome-wide association tests** |  |  |  |
| EW_LoF_skat | MetaSKAT | Functional exome-wide rare variant SKAT tests for MAF<1% (loss-of-function) | 3,208 (9,113) |
| EW_LoF_skat-o | MetaSKAT-O | Functional exome-wide rare variant SKAT-O tests for MAF<1% (loss-of-function) | 3,208 (9,113) |
| EW_functional_skat | MetaSKAT | Functional exome-wide rare variant SKAT tests for MAF<1% (loss-of-function and missense) | 14,909 (256,733) |
| EW_functional_skat-o | MetaSKAT-O | Functional exome-wide rare variant SKAT-O tests for MAF<1% (loss-of-function and missense) | 14,909 (256,733) |
| EW_naive_skat | MetaSKAT | Naïve exome-wide rare variant SKAT tests for MAF<1% | 50,717 (1,783,548) |
| EW_naive_skat-o | MetaSKAT-O | Naïve exome-wide rare variant SKAT-O tests for MAF<1% | 50,717 (1,783,548) |
| **Genome-wide association tests** |  |  |  |
| GW_skat | MetaSKAT | Genome-wide rare variant SKAT tests for MAF<1% | 1,845,982 (35,858,684) |
| GW_skat-o | MetaSKAT-O | Genome-wide rare variant SKAT-O tests for MAF<1% | 1,845,982 (35,858,684) |

**Appendix 3. Description of statistical tests implemented**

The *cohort* *arm* of the UK10K (uk10k-cohort) study aimed to assess the contribution of genetic variation genome wide to a range of quantitative traits in 3,781 healthy individuals from intensively studied cohorts of European ancestry, namely the Avon Longitudinal Study of Parents and Children (ALSPAC) ^6^ and TwinsUK ^7^. Whole genome sequence (WGS) at 7x read depth was employed as a method maximising total variation detected whilst allowing access to noncoding variation where most GWAS signals lie. The final uk10k-cohorts call set contained over 42M single nucleotide variants (SNVs), of which 34.2M were rare (minor allele frequency [MAF]<1%) and 2.3M were low-frequency (MAF 1-5%) and ~3.5M INDELs. A total of 64 different phenotypes were used for analysis, including traits of primary clinical relevance in 11 major phenotypic groups (obesity, diabetes, cardiovascular and blood biochemistry, blood pressure, dynamic measurements, birth, heart, lung, liver and renal function; **Appendix 1**). Of these, 31 phenotypes were available in both studies, 19 were unique to TwinsUK and 14 were unique to ALSPAC. Different testing strategies were employed for analysis (**Appendix 2**), which generated overall 1,460 million individual association statistics.

**Single-variant tests.** We fitted linear models on standardised traits, residualised for relevant covariates, to test associations of allele dosages with 13,074,236 SNVs and 1,122,542 biallelic INDELs (MAF≥0.1%) and 18,739 large deletions in whole-genome sequenced samples.

**Rare variant tests.** For each of the four scenarios below, we applied two separate statistical models with different properties to rare variants (MAF<1%): sequence kernel association tests (SKAT) and burden tests implemented in SKAT and SKAT-O ^9,10^. SKAT is a variance-component multiple regression test which retains power in settings where neutral variants or variants with opposite direction of effects could result in loss of power. SKAT-O represents the best linear combination of SKAT and burden tests, which is supposed to maximize power. Analyses were either exome-wide, or genome-wide. For *exome-wide* rare variants tests, we focused on variants in coding exons and UTR regions of genes, with three different variant selection strategies. (i) *Naïve* tests considered all variants in exons, untranslated regions (UTRs) and essential splice sites, where all variants were given equal weight of being causal. *Functional* tests considered missense and loss-of-function (LoF) variants, the latter predicted to cause essential splice site donor or acceptor changes, stop codon gains and frameshift mutations. Finally, tests including only *loss-of-function* variants were carried out. For *genome-wide* analyses, we partitioned the genome into 3kb half-overlapping tiling windows with an average of 37 variants per window (median=39 variants). In total, for each trait, we generated SKAT and SKAT-O p-values and their corresponding MetaSKAT p-values for more than 1.8 million windows across the genome.

For 31 overlapping traits (‘core’ traits) with phenotype data in both ALSPAC and TwinsUK, associations were run independently in the two cohorts and meta-analysed using inverse variance models. In the browser we report the association p-values (-log10 scale) for the meta-analysis of the two studies. For traits with data available in only ALSPAC or TwinsUK, we report association statistics for that study.

**Appendix 4. Genome Browser Functionality**

The Biodalliance Genome Browser embedded on the UK10K website can be used to interactively explore the UK10K association results and produce high quality publication images. A typical starting view (**Figure 1**) shows the genome track on top followed by a number of selected genome data tracks. An overview of the genome browser commands is shown in **appendix 5**. In the following sections we describe the functionality of the Biodalliance genome browser. This includes navigation around the tracks, selecting tracks to display or adding new data. We also describe how to display LD information and produce high quality images from the current track view.

**Requirements.** The Biodalliance Genome Browser can be accessed using the latest versions of most major web browsers (Chrome, Firefox, Internet Explorer and Safari).

**User Interface Overview.** The user interface can generally be divided into three regions, the top bar, the main display area and a sidebar area (which collapses while not in use). The top bar fulfills two functionalities. First, it provides functionality for navigating around the track view. Second, on the right side it holds buttons for accessing a number of additional sidebars. The different sidebars are activated by clicking on the respective buttons and are used for showing additional information, editing settings or managing the displayed data. The main display area is used for visualizing the genome track data.

**Managing Displayed Tracks**

The Add-sidebar holds various options to show/hide, add/remove tracks to the track hub. In general, available data tracks are organized in tab categories. A chosen track from a tab category is displayed or hidden according to the checkbox in front of the track name.

The UK10K genome browser comes equipped with tab categories holding the UK10K association result tracks.

**UK10K Tracks**. There are nine tab categories holding UK10K association result tracks (single_variant, EW_LoF_skat, EW_LoF_skat-o, EW_functional_skat, EW_functional_skat-o, EW_naive_skat, EW_naive_skat-o, GW_skat, GW_skat-o). The track naming scheme is summarised in **Appendix 2.**

**Adding Custom Data**. Genomic data in range of supported formats (including bigWig, bigBed, plain bed and wig, BAM and VCF), which are stored on another webserver or on the local hard-drive can be added to the browser via the “Binary”-tab. Data served using the DAS protocol (<http://biodas.org/>) is also supported.

**Connecting UCSC Track Hubs.** The Biodalliance genome browser has support for connecting to UCSC browser “track hubs”. To connect an existing UCSC track hub, select the +-tab, specify the URL of the “hub.txt” file and click the add-button.

**Navigation Options**

The user is provided with various methods for navigating through the association results data tracks.

1. *Direct Navigation*. The top bar of the browser view holds a text field for direct navigation. Initially, the text field shows the displayed region of the genome. To navigate to a different region, the user can type a genome region (e.g. “16:29,993,244..30,023,244”) or genome position (e.g. “16:29,993,244”) and navigate there by pressing [Enter]. It is also possible to search for rsIDs (e.g. type “rs10”) or genes (e.g. type “CETP”) in the same way.
2. *Scroll Navigation*. To scroll navigate through the track data, the user can either use the keyboard arrow keys, horizontal trackpad scroll or drag the view with a mouse click. For convenience, the navigation direction can be inverted in the browser settings.
3. *Threshold-leap* *Navigation*. The Biodalliance genome browser allows to search a quantitative track for features scoring above a given threshold value. Search in upwards or downwards direction is initiated by clicking the arrow buttons to the left and right of the top bar (or press Ctrl+LeftArrow / Ctrl+RightArrow). The threshold value can be adjusted via the track settings sidebar (or press Shift+UpArrow / Shift+DownArrow).

**Zooming**. It is possible to increase or decrease the size of the displayed genomic region. To zoom in or out the user can either use the zoom slider in the top bar (or press + / -). In addition, a base resolution zoom can be toggled by pressing the [Space].

**Edit Settings.** The Biodalliance Genome Browser allows for minor configuration changes to respond to user inputs via the configuration sidebar. Also, the track editing sidebar allows for adjusting track settings individually for each track.

**Biodalliance Settings**. The configuration sidebar holds options to invert scrolling directions. The user can also choose the horizontal position of the vertical score guideline or turn it off completely. The Reset button can be used to reset settings to default.

**Track Settings**. Each displayed track in the track view can be configured to some extent by using the track settings sidebar. The sidebar holds options to change the track name, color, height or style (histogram, line plot, ribbon, scatter). Furthermore, the displayed horizontal value range can be adjusted and a threshold value for threshold navigation can be specified.

**Visualisation options**

**Merge Tracks**. For visualization purposes, multiple tracks can be merged/overlayed into a new single track. To do so, first select the tracks to be merged (hold Shift to select multiple tracks) , then press “ctrl + m”. A new track will appear showing an overlay of the selected tracks.

**Image Export**. The Biodalliance genome browser supports to export a track view as a scalable vector graphic (SVG) file. To create and export the SVG file showing the current track view, select the printer-button [icon], which toggles the image-export-sidebar and click the export button. A download link to the created SVG file will appear.

**Feature Info Box**. When clicking on a track feature a feature info box with additional information about the clicked feature pops up. For UK10K single point trait tracks this box holds fields like rsID, effect allele, effect allele frequency, VQSLOD score and indicators if the SNP is discovered in HapMap or 1000Genomes.

**SNP Consequence Symbols**. SNP consequences are indicated by the symbol displayed for that SNP. By default, SNPs are displayed as a circle, whereas squares indicate an untranslated region SNP, upwards pointing triangles indicate a splice region SNP, downwards pointing triangles indicate a missense region SNP and stars indicate a regulatory region SNP.

**LD Calculations**. The UK10K genome browser supports to display linkage disequilibrium scores with respect to a selected reference SNP. LD scores (r^2^) are indicated using an LD Score coloring scheme (described in the user documentation), whereas each SNP is coloured according to its LD score. To calculate LD scores with respect to a chosen reference SNP, click on the reference SNP to open the SNP info box and then click the “Make ref.”-button. Once the LD scores have been calculated SNP colors are updated according to their LD scores. Multiple references (maximum of 5) can be created by making the first SNV reference and then by selecting and adding extra SNVs via the “Add ref.” button- note that this changes the LD colour scheme, also detailed in the user documentation.

**Appendix 5. Reference summary of tests implemented in the UK10K cohorts data and key navigation functions**


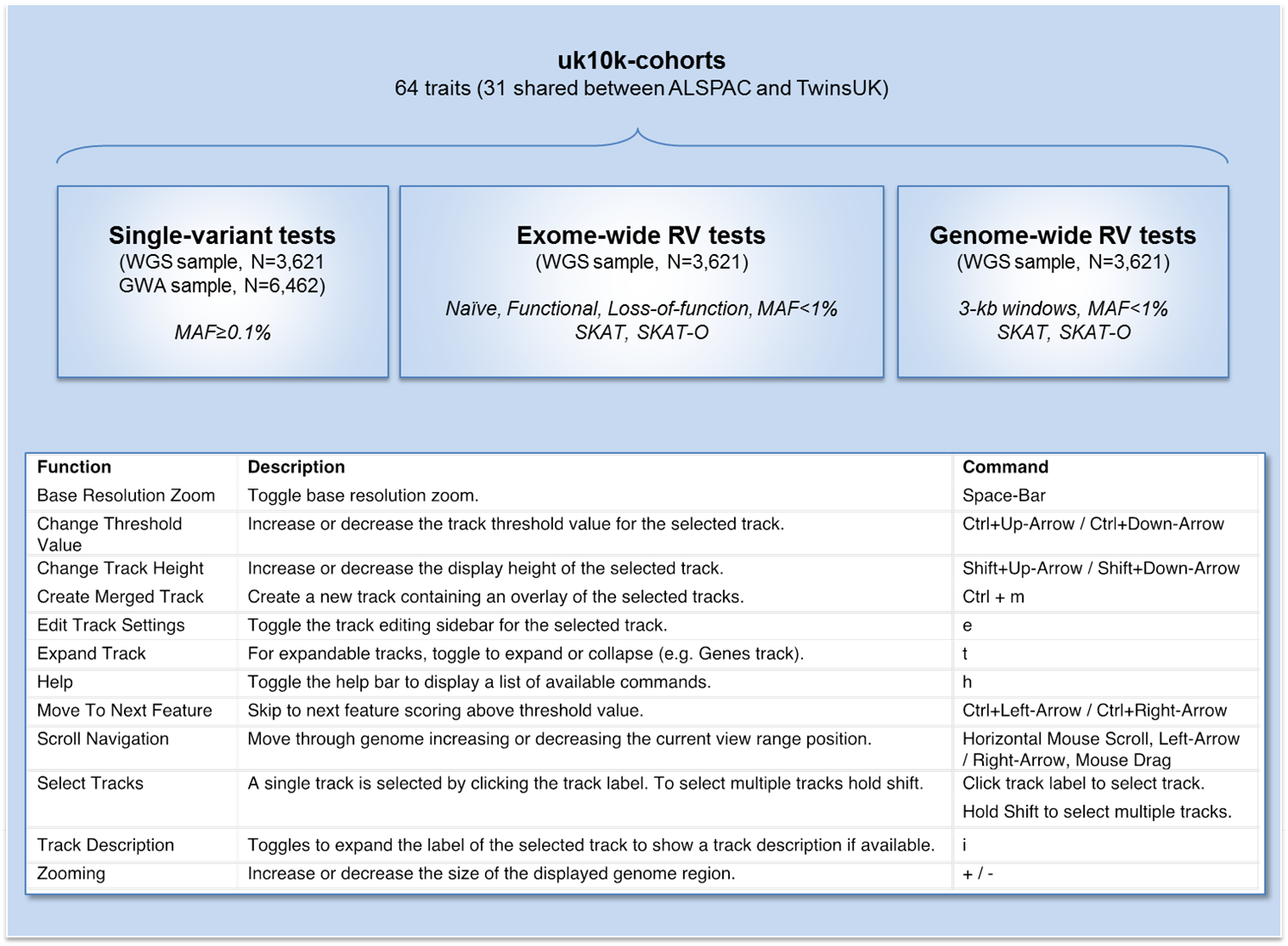

Supplement: Supplementary Data [file supp_btv491_UK10K_browser_suppl_for_upload.docx]
